# Social Determinants of Rural Household Food Insecurity under the Taliban Regime

**DOI:** 10.3390/nu15071681

**Published:** 2023-03-30

**Authors:** Wasiuddin Najam, Temitope Ibiyemi, Sajia Aziz, Rafiuddin Najam, Wanjiku N. Gichohi-Wainaina, Wilna Oldewage-Theron

**Affiliations:** 1 Department of Nutritional Sciences, Texas Tech University, Lubbock, TX 79409, USA; 2 School of Public Health, University of Illinois Chicago, Chicago, IL 60612, USA; 3 School of Public Policy, Oregon State University, Corvallis, OR 97331, USA; 4 Department of Sustainable Food Systems and Development, University of the Free State, Bloemfontein 9300, South Africa

**Keywords:** food insecurity, rural households, Afghanistan, Taliban

## Abstract

Despite the severity of food insecurity in Afghanistan, little is known about the factors contributing to household food insecurity (HFI) under the Taliban regime. Therefore, this paper investigated the social determinants of severe HFI in rural areas of Afghanistan. We used the fifth-round survey of 6019 rural households from 25 provinces, collected between July and August 2022 by the Food and Agriculture Organization. We used binary logistic regression to examine the association between household characteristics and HFI. The majority of household heads were male (97.8%) with no education (62.8%). The findings showed that female-headed households had significantly higher odds of severe HFI. Household heads with any level of formal education had significantly reduced odds of severe HFI, while the odds of severe HFI was not different among those with religious/informal household-head education compared to those with no education. Likewise, engagement in any type of agricultural activity decreased the odds of severe HFI. Additionally, household income per member was negatively, while household size was positively associated with severe HFI. In summary, interventions to alleviate HFI among rural households should prioritize income-generating opportunities and skills targeting households with female heads, low levels of household-head education, larger size, no agricultural activities, and low income.

## 1. Introduction

A household suffers from food insecurity when the household members are unable to consistently access safe, nutritious, and sufficient food that is essential for maintaining a healthy and active lifestyle [1]. Food insecurity is a significant global challenge, affecting millions of individuals and households globally [2]. The global prevalence of moderate or severe food insecurity was approximately 2.3 billion people in 2021, which is about 29.3% of the world population, an increase of an additional 350 million individuals since 2019 [2]. Several factors, including political instability [3], climate variability and extremes [4], and downturns of the economy [5], as well as the COVID-19 pandemic that further exacerbated the food insecurity situation, have contributed to the high prevalence of global food insecurity [2].

Food insecurity has been a major issue in Afghanistan for decades [6,7]. Food availability and access have been impacted by multiple factors, and poor nutrition indices indicate challenges in the utilization pillar of food insecurity; however, the stability pillar has been a particularly strong pillar that has influenced food insecurity in Afghanistan [6]. Prior to the year 2000, the prolonged political instability and conflict in Afghanistan negatively affected food production, supply, and access across the country, which contributed to the high prevalence of food insecurity among Afghans [6]. As a result, the country suffered “extremely alarming hunger” in 2000 based on the Global Hunger Index [7]. This situation was addressed to some extent by the enormous support from various countries and international organizations following the fall of the Taliban regime in 2001 and the rise of a new government [8]. Nonetheless, the Afghan people have continued to suffer from a persistently high rate of food insecurity and hunger [7,9]. In the years after 2014, following the completion of the United States of America (USA)-led coalition combat mission in Afghanistan [10] along with the political transition, political turmoil and insecurity increased in the country. This situation negatively impacted the food security status in the country, as evidenced by the three-year average prevalence of moderate and severe food insecurity that increased from 45.1% between 2014 and 2016 to 70% between 2019 and 2021 in Afghanistan [9].

After almost 20 years of conflicts, on 15 August 2021, the Taliban regained power once again [11]. This led to the withdrawal of international forces, disruption, and closure of international organizations, a decline in monetary aid, and introduction of sanctions such as freezing the Afghanistan government’s assets to the Taliban regime by the USA [12], which led to a high unemployment rate and decreased household income, particularly among individuals with formal education [13,14]. These conditions have further exacerbated the challenge of food insecurity, mainly household food availability and access [14]. As a result, the food insecurity status has worsened in the country, with nearly 19 million people (47% of the population) experiencing high levels of acute food insecurity between September and October 2021, compared to the 11.15 million people (36% of the population) between August and October 2020 [15].

The majority of the Afghan population resides in rural settings, where agricultural activities are the main source of income [16,17,18]. Therefore, in addition to political instabilities and disruption in economic activities, environmental factors (e.g., drought, plant diseases, flood, earthquake) also contribute to the increasing prevalence of food insecurity in Afghanistan [16,19,20,21,22]. According to recent reports, in 2022, 83% and 96% of rural farmers reported reductions in the planted area and agricultural production, respectively, mainly due to lack of water and plant diseases [16]. Since agricultural activities are one of the main income sources for the majority of Afghan rural households, natural disasters cause higher financial stress and lower total income, which may exacerbate the already high prevalence of food insecurity [16].

Currently, Afghanistan is suffering one of the most catastrophic humanitarian crises in the world, with 47% of the population facing high levels of acute food insecurity [15]. In particular, 84% of rural households were facing moderate and severe food insecurity in 2022, according to the Food and Agriculture Organization (FAO), which utilized the Food Insecurity Experience Scale (FIES) [16]. The prevalence of food insecurity is reported to be higher among female-headed households [14]. The Taliban’s decisions, particularly the ban on women’s right to work and attend institutions of secondary and higher education, violates basic human rights and has led to the suspension and interruption of several non-governmental organizations’ operations, which were critical in delivering aid such as monetary and food assistance and services to communities in Afghanistan [23]. As a result, many people lost their source of income, which will deepen the poverty rate and magnify the prevalence of food insecurity for years to come.

Interventions are needed to improve household living conditions, particularly access to food, to address the existing crisis in Afghanistan. Hence, it requires a comprehensive understanding of the theoretical foundation for developing interventions [24], which includes understanding the characteristics of the population that the intervention is targeted to, as well as other key factors that may impact implementation success, as noted by the Consolidated Framework for Implementation Research (CFIR) [25]. Therefore, understanding the significant factors that increase the likelihood of food insecurity is of utmost importance to develop effective interventions and policies aimed at improving food insecurity status.

Globally, previous studies demonstrated that living in low-income countries, rural areas, larger households, and being female, having low education levels, and low income levels increase the likelihood of experiencing food insecurity [26,27,28]; however, participating in agricultural activities, in countries where agriculture is considered the main source of income, can reduce the chance of experiencing food insecurity [29,30]. Similar trends were observed in previous studies conducted in the rural areas of Afghanistan prior to the Taliban takeover in 2021 [31,32]. They suggested that households with a female head and larger size were negatively, while education attainment, income and involvement in agricultural activities were positively associated with household food security [31,32].

While the recent crisis of food insecurity in Afghanistan has received increased attention from the international community, the factors that play a significant role in HFI after the Taliban takeover remain poorly understood. Hence, we aim to fill this gap by examining social determinants (household-head gender, head education, size, agricultural activity, and income per member) of severe HFI in rural areas of Afghanistan to assist the international community and policymakers in effectively targeting vulnerable populations during the ongoing crisis.

## 2. Materials and Methods

### 2.1. Study Design

This study used the data from the fifth-round household survey of the Data in Emergencies (DIEM)-Monitoring system in Afghanistan, which were collected cross-sectionally between 23 July and 26 August 2022 [33].

### 2.2. Data in Emergencies Monitoring System

The DIEM-Monitoring system was established under the Food and Agriculture Organization (FAO) of the United Nations. The main purpose of the DIEM-Monitoring system is to collect data from households and key informants in countries prone to multiple shocks [34]. Therefore, the DIEM-Monitoring system conducts household surveys in Afghanistan to monitor agricultural livelihoods and food security.

### 2.3. Study Population, Setting, and Data Collection

**Figure 1:**
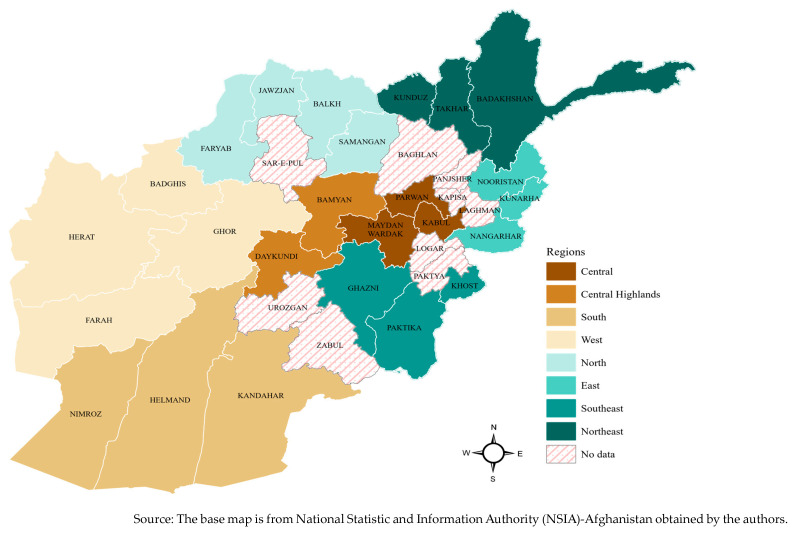
Provinces within the regions of Afghanistan included in the study sample.

The DIEM methodology was published previously [16]. The sample included rural households that were randomly selected using two-step cluster sampling and probability proportional to size. The total number of households interviewed was 6019, which is a representative sample of rural households in 25 out of 34 provinces that include all 8 regions of Afghanistan (Figure 1). The DIEM data captured household-level information with face-to-face interviews among rural households using the Kobo Toolbox. The Kobo Toolbox is a toolkit used to collect and manage data in challenging environments and humanitarian emergencies such as the current situation in Afghanistan.

### 2.4. Measures

#### 2.4.1. The Food Insecurity Experience Scale

The outcome used in this study was HFI status. The FIES was used to measure the food insecurity status of households in the 30 days prior to data collection [35]. The FIES was developed by the FAO for measuring the severity of food insecurity as a latent trait due to the lack of money or other resources. Table 1 represents the FIES questionnaire, which consists of 8 questions with binary yes or no response options, and each question belongs to either a mild, moderate, or severe category of food insecurity. The total FIES score is 8, for which an affirmative and a negative response receive a value of 1 and 0, respectively. The Voices of the Hungry project of the FAO suggested that the raw score can be divided into four categories: food secure (0 score), mildly food insecure (1–3 score), moderately food insecure (4–6 score), and severely food insecure (7–8 score) [36].

#### 2.4.2. Covariates

Sociodemographic information was collected using the household level questionnaire in face-to-face interviews. Although there are various social determinants associated with HFI, the determinants provided by the DIEM dataset include household-head gender, head education, size, agricultural activity, and income. Household-head gender was categorized as male or female, and household-head education level was recorded as none/did not complete primary school, completed primary school, completed secondary school, completed higher education (university/college) degree, or religious or informal education. Likewise, household agricultural activity was grouped into 4 categories (none, livestock production, crop production, both livestock and crop production). Household size was recorded in numbers. Finally, the total household income was reported, and to estimate the household income per member, we divided the total household income by total household size.

### 2.5. Statistical Analysis

The *R* language within the R-Studio environment (version 2022.12.0+353) was used to perform all the statistical analyses. To objectively verify the validity and reliability of the FIES data, the Rasch model was implemented using the “RM.weights” package. The Rasch model helps ensure that the FIES is a valid and reliable measure of food insecurity and provides a way to objectively evaluate the scale’s properties and its ability to accurately capture the experiences of those facing food insecurity [35]. The weighted infit statistics from the Rasch model analysis were used to assess the validity and reliability of the FIES in measuring food insecurity. The ideal infit statistic is 1.0, with a broadly acceptable range of 0.7–1.3 [35]. The weighted infit statistics for the FIES data in our study were within the acceptable range of 0.7–1.2, which verified that the FIES is a reliable and valid measure to assess food insecurity in this population. To estimate the prevalence of food insecurity, we have used the deterministic classification, in line with the FAO’s Voices of Hungry recommendation [37].

All the analyses were conducted using the “Survey” package and accounted for the complex sampling design and weight to obtain population-representative findings. The household income per member was transformed by taking the natural logarithm of the household income per member plus one, which accounts for taking the logarithm of values that are equal to zero, to normalize the right-skewed distribution of data. The household size was presented as mean and standard error (SE), while the household income per member was presented as mean ± SE and median (Q1–Q3). The categorical data were presented as proportion and SE. To provide a comprehensive understanding of the determinants of severe HFI, univariate and multivariate logistic regression models were used. For all regression analyses, household-head gender, head education, agricultural activity, size, and the natural log of household income per member were used as independent variables. Based on the FAO recommendation for using the FIES as a dependent variable [36], each household was assigned to one of the two categories of food insecurity according to their FIES raw score: 0 = food secure to moderate HFI (0–6 score) and 1 = severe HFI (7–8 score). The generalized variance inflation factor (VIF) [38], which takes into account the degrees of freedom, demonstrated no multicollinearity in the multivariate logistic regression model. The logistic regression coefficients are represented as odds ratios (95% confidence interval) [OR (95% CI)], and a *p* value < 0.05 was considered statistically significant.

## 3. Results

### 3.1. Households Characteristics

The household characteristics of rural households are summarized in Table 2, which indicates that the households were predominantly male-headed (97.8%), and 62.8% of household heads had no education or did not complete primary school. Additionally, on average, the rural households had 9.5 members. In terms of agricultural activities, 13.1% and 46.6% of the households participated in livestock and crop production, respectively, while 27.2% had both crop and livestock production. Shockingly, 22% of households reported no income, and the mean household income per member was AFN 2021 (USD ~22.7) in the past three months.

### 3.2. Food Insecurity Experience Scale

**Figure 2:**
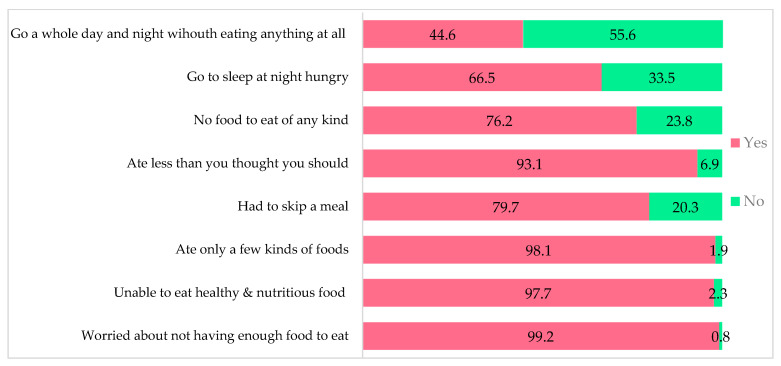
Weighted percentages of responses to the FIES questions.

Figure 2 represents the weighted proportion of responses to the FIES questionnaire. Alarmingly, every household (99.2%) reported that they worried about not having enough food to eat. Likewise, 76.2% reported not having any food to eat, while 65.53% and 44.6% indicated that they sleep hungry at night and went an entire day and night without eating anything, respectively. The prevalence of food insecurity based on deterministic classification demonstrated that 95% of the rural households experienced moderate or severe food insecurity, while severe food insecurity was reported by 63% of the households.

### 3.3. Social Determinants of Household Food Insecurity

#### 3.3.1. Univariate Analysis of Social Determinants of Severe Household Food Insecurity

Table 3 shows the univariate logistic regression results for the social determinants of severe HFI. The findings suggest that female-headed households had higher odds [OR (95% CI) = 2.67 (1.56–4.56)] of severe HFI than male-headed households. Household heads with primary or secondary education levels had lower odds of severe HFI relative to those with no education/incomplete primary education; however, those with college/university education or religious/informal education are not significantly different compared to the reference group. Households with any forms of agricultural activities had lower odds of severe food insecurity compared to households with no agricultural activities. Finally, the natural log of household income per member in the past three months was a significant predictor of severe HFI [OR (95% CI) = 0.91 (0.88–0.93)].

#### 3.3.2. Multivariate Analysis of Social Determinants of Severe Household Food Insecurity

Table 4 represents the results of region-adjusted social determinants of severe HFI from a multivariate logistic regression model. The results indicated that the odds of severe HFI is higher among female-headed households. Likewise, as household size increased by one additional member, the odds of severe food insecurity increased by 1.03 (1.004–1.06) times. Moreover, our findings indicate that if the household head had any level of formal education, the odds of severe food insecurity were reduced compared to those with no education/did not complete primary school. Engagement in any form of agricultural activities reduces the likelihood of severe food insecurity in a household, compared to those with no agricultural activities. Finally, similarly to the results reported in the univariate model, the natural log of household income per member in the past three months was a significant predictor of severe HFI [OR (95% CI) = 0.92 (0.89–0.95)].

## 4. Discussion

In this study, the majority of households are headed by males (97.8%), among which 62.8% are uneducated. Rural households are mainly involved in agricultural activities, with 46.6% of households engaged in crop production. Also, one out of five rural households (22%) reported no income generation in the past three months prior to data collection. Moreover, our findings suggest that more than nine out of ten households (95%) living in rural areas suffer from moderate or severe levels of food insecurity, while severe food insecurity was reported by six out of ten (63%) rural households.

According to the FAO, prior to the Taliban takeover, the three-year average prevalence of moderate and severe food insecurity in Afghanistan was 70% between 2019 and 2021 [9]. The evidence suggests that the prevalence of food insecurity increased since 2021. In October 2021, the Integrated Food Security Phase Classification (IPC) reported that 47% of Afghans were experiencing acute levels of food insecurity, which increased from 36% in October 2020, and 35% in May 2020 [15]. Likewise, according to the World Food Programme (WFP), the rate of inadequate food consumption in Afghanistan increased markedly, from 81% just before 15 August 2021, to 97% in October 2021, and it remained between 92% and 98% from September 2021 until May 2022 [40]. Considering the high prevalence of food insecurity in Afghanistan, this study aimed to assess the social determinants of rural HFI, with specific focus on household-head gender, head education, size, agricultural activity, and income, one year after the Taliban returned to power.

### 4.1. Household-Head Gender

Our findings shows that female-headed households had higher odds of experiencing severe HFI than male-headed households. The findings align with other studies conducted in developing countries around the world [41,42,43,44]. Similarly, to our findings, a recent study by Santos et al. 2022, in a low-income district of Lima, Peru, showed that female-headed households had higher odds of food insecurity relative to male-headed households [43]. A systematic review and meta-analysis of 141 articles by Negesse and colleagues from Ethiopia, a country facing dire food insecurity due to economic instability, conflict, drought, and extreme poverty, just like Afghanistan, also reported that female-headed households in Ethiopia also had higher odds [OR (95% CI) = 1.94 (1.26–3.01)] of suffering from food insecurity compared to male-headed households [42]. A growing body of evidence in different parts of the world suggests that female-headed households are more susceptible to food insecurity due to gender-based discrimination [42,43,44,45,46,47,48,49]. This can be explained by the limited access for women to income-generating resources, education, and skills, and they are more likely to be vulnerable to major shocks and disasters (drought, flood, wars, and economic meltdown) [45,48,49].

Prior reports in Afghanistan indicated that female-headed households were more likely to experience frequent food insecurity than male-headed households [50,51]. There have been increased restrictions imposed on women in the country in the wake of the Taliban regime [52]. Women face more stringent regulations with respect to securing jobs outside the home and inter-city travel with male chaperones, and have been subjected to more gender-based discrimination [52,53]. These events have threatened the food security status of women and female-headed households in Afghanistan. Hence, given the existing conditions, female-headed households are more vulnerable to food insecurity than households headed by males.

### 4.2. Household-Head Education

Our findings demonstrated an inverse association between household-head education and severe HFI status. Compared to those with no education/did not complete primary school, those who completed primary, secondary, and college/university education had reduced odds of experiencing severe HFI. There is well-established evidence from numerous studies that households with more educated heads are less likely to suffer from food insecurity in developing and low-income countries [26,27,28,29,31,32,43,54,55]. This might be because the educational attainment of household heads is associated with more job opportunities, increased skill development, higher off-farm income, improved sanitation, nutrition knowledge, and health decisions, all of which can improve household food security status [55,56,57]. Furthermore, increased household-head education can improve agricultural productivity and income [55,56]. Most of the households in our study are involved in agricultural activities, and previous research has also suggested that educated farmers are more likely to utilize advanced farming technologies and strategies and diversify their income-generating sources in a time of need, which advances the food security status of households [55,56,58,59].

Our study did not find a relationship between household-heads with religious or informal education and experience of severe HFI, despite the reported favoritism in the allocation of monetary aid, job opportunities, and other income-generating activities toward Taliban supporters, many of whom are people with informal religious education [60,61]. However, we opine that it is also possible that respondents with religious education may under-report their level of food insecurity, due to their strong religious beliefs, and consider it a social stigma, which might explain the non-significant findings among this population. Previous studies also suggest that people with religious beliefs may find it challenging to acknowledge the feeling of anxiety or worry in their lives, as they attribute events, whether positive or negative, to the supreme power of God [62,63].

### 4.3. Household Size

Afghanistan has a higher-than-global-average household size [64], and multigenerational households, in which adult children live with their parents or in-laws throughout their life, are common [65]. Our findings indicate a positive association between household size and severe HFI. Other studies among developing countries in Sub-Saharan Africa, South Asia, and the Middle East have reported similar results [31,43,56,66]. In Pakistan, Shahzad and co-authors denoted that having a large family size is a significant predictor of food insecurity among low-income households in Punjab [41]. Likewise, another study by Ahmadzai and Aryobi (2021) among rural households in the Paktia province of Afghanistan found that household size is a significant predictor of food security status, where larger households are more likely to be food insecure than those with fewer members [31]. A common challenge of food-insecure households is the distribution of insufficient and limited food. Often, household food intake is distributed based on the gender, age, and dietary needs of the youngest in the household [67,68]. Thus, an increase in household size introduces an extra burden on food consumption and food security status.

### 4.4. Household Agricultural Activity

Food security status was positively influenced by household agricultural activity in our study. Households engaged in agricultural activity in the form of crop, livestock, or both crop and livestock production are less likely to experience severe HFI. This finding is similar to several studies that have shown that engaging in agricultural activity can improve food security status in developing countries [29,69,70,71,72]. A study by Pawlak and Kolodziejczak (2020) found that increasing agricultural activities is a key driver of improving food availability and access in rural areas of developing countries [29]. Another study in rural communities of Madagascar found that the likelihood of household food insecurity decreased with increased agricultural activity [71]. Our study findings might be attributed to the fact that engaging in agricultural activity can improve food security by increasing production and availability of foods, increasing income, and reducing poverty [29,69].

Our finding is also supported by a secondary analysis of households in Northern Ghana [69], which is one of the nation’s poorest regions. Danso-Abbeam et al. (2021) revealed that engaging in both crop and livestock production improved household food security status [69]. In addition to providing income variety for farmers engaged in mixed farming, the evidence has shown that livestock production can decrease the risks associated with crop production [72,73,74]. Livestock production also provides a buffer against climatic and economic instability [70,75]. Hence, engaging in any form of agricultural activity favors households in Afghanistan to be more food secure, relative to those with no agricultural activities.

### 4.5. Household Income

Poverty is a direct factor that impairs access to food and leads to food insecurity [66]. Poor households have limited and restricted ability to purchase the needed variety of foods to support a healthy and active life [31]. Our study also revealed that a one-unit increase in the natural log of household income per capita in the past three months was associated with an 8% reduction in the odds of severe HFI. The findings are consistent with previous studies conducted in low and middle-income countries, which show that poor households with reduced income have limited food access and are more susceptible to food insecurity [28,31,67,76,77]. Before the Taliban takeover, a study in the Paktia province of Afghanistan found that household income was the most significant predictor of food security status among rural households [31]. Another study among farming households in the Takhar province of Afghanistan found that both household farm income and non-agricultural income significantly positively influenced the food security status of respondents [32].

The economic situation in Afghanistan deteriorated after the Taliban takeover, impairing household income [12,60,78]. Before the Taliban takeover, monetary aid from the international community accounted for a large percentage of the government budget, [79,80] which enabled the former Afghan government to provide services in hard-to-reach areas; however, under the Taliban government, such monetary aid, for the most part, has been reduced or abandoned due to sanctions on the Taliban regime [12,79]. Furthermore, hundreds of thousands of workers lost their jobs, and the country’s currency depreciated [78,81]. There has also been a hike in food and fuel prices, which further exacerbates the food insecurity rate in the country [12]. Inflation, particularly food inflation, reached a 14-year record high in June/July 2022 [82]. A recent report by the World Bank showed that food prices increased by 24.9% in July 2022, compared to July 2021 [83]. Likewise, compared to the prices a year ago, in May 2022, the WFP reported a 45% increase in wheat, a 49% increase in wheat flour, and a 20% increase in rice prices, some of the country’s common staple foods [15].

### 4.6. Strengths and Limitations

This study has several strengths. First, it featured a large nationally representative sample that was randomly selected from rural households in Afghanistan. Second, this is the first study, to our knowledge, examining how the investigated social determinants are associated with severe HFI in Afghanistan after the Taliban regained power in 2021. This will provide valuable insights for international organizations aiming to address HFI in Afghanistan. Lastly, univariate and multivariate analyses were conducted to provide a better understanding of the food insecurity determinants. The cross-sectional design of the study, which provides information related to a particular time of the year, might be considered a limitation. Another limitation could be the low proportion of female-headed households in the study sample, as results cannot be generalizable to all female-headed households. However, the randomly selected nature of the sample allows us to rectify this concern, which demonstrates a similar proportion of female-headed households to previous studies in rural areas of Afghanistan [31,32]. Furthermore, Afghanistan is a male-dominated society, where males are generally the household head [65].

## 5. Conclusions

Food insecurity remains a major concern among rural households in Afghanistan, with 63% experiencing severe HFI based on deterministic classification of FIES. Our findings suggest that all investigated social determinants, including household-head gender, head education, size, agricultural activity, and income are associated with severe HFI. Households headed by people with formal education, along with households involved in agricultural activity, and those with higher income per family member are less likely to suffer from severe HFI. However, female-headed and larger-sized households are more likely to experience severe HFI.

Interventions and strategies by international organizations to alleviate severe food insecurity among rural households should prioritize income-generating opportunities and skills targeting households with female heads, low levels of household-head education, larger numbers of members, no agricultural activity, and low income. The consistency of our results with prior studies highlights the relevance of successful interventions in other contexts that could be adapted to address the ongoing crisis in Afghanistan.

## Figures and Tables

**Table 1:** FIES Questionnaire.

	Questions	Severity of Food Insecurity
1	During the last 30 days, was there a time when you or others in your household were worried about not having enough food to eat because of lack of money or other resources?	Mild
2	During the last 30 days, was there a time when you or others in your household were unable to eat healthy and nutritious food because of lack of money or other resources?	Mild
3	During the last 30 days, was there a time when you or others in your household ate only a few kinds of foods because of lack of money or other resources?	Mild
4	During the last 30 days, was there a time when you or others in your household had to skip a meal because of lack of money or other resources to get food?	Moderate
5	During the last 30 days, was there a time when you or others in your household ate less than you thought you should because of lack of money or other resources?	Moderate
6	In the past 30 days, was there ever no food to eat of any kind in your house because of lack of resources to get food?	Moderate
7	In the past 30 days, did you or any household member ever go to sleep at night hungry because there was not enough food?	Severe
8	In the past 30 days, did you or any household member ever go a whole day and night without eating anything at all because there was not enough food?	Severe

**Table 2:** Weighted characteristics of the households.

Characteristics		Overall (N = 6019)	
		Mean	SE
Household-Head Gender (%)	Male	97.8	0.003
Household-Head Education (%)	None/Did not complete primary school	62.8	0.0098
	Completed primary school	13.1	0.006
	Completed secondary school	10.7	0.007
	Completed college/university	9.5	0.007
	Religious/informal education	3.8	0.003
Household Size Range		9.5	0.077
Household Agricultural Activity (%)	No Agricultural Activity	13.2	0.007
	Livestock Production	13.1	0.008
	Crop Production	46.6	0.011
	Crop and Livestock Production	27.2	0.009
Household Income per Member in past 3 months *	Mean (SE)	AFN 2021 (USD ~22.7)	50.43
	Median (Q1–Q3)	AFN 1500 (USD ~16.8)	583–2857

* Afghani (AFN) to United State dollar (USD) is based on the conversion rate on 1 August 2022 [39].

**Table 3:** Univariate analysis of social determinants of severe household food insecurity.

Variables		Severe Household Food Insecurity	
		OR (95% CI)	*p* Values *
Household-Head Gender	Male	1	
	Female	2.67 (1.56–4.56)	0.0003
Household-Head Education	None/Did not complete primary school	1	
	Completed primary school	0.63 (0.50–0.78)	<0.0001
	Completed secondary school	0.60 (0.47–0.78)	0.0001
	Completed college/university	0.77 (0.57–1.04)	0.09
	Religious/informal education only	1.02 (0.74–1.41)	0.89
Household Size		1.04 (1.01–1.06)	0.004
Household Agricultural Activity	No Agricultural Activity	1	
	Livestock Production	0.44 (0.31–0.62)	<0.0001
	Crop Production	0.47 (0.35–0.63)	<0.0001
	Crop and Livestock Production	0.42 (0.31–0.58)	<0.0001
Natural Log of Household Income Per Member in Past 3 Months		0.91 (0.88–0.93)	<0.0001

*p value < 0.05 was considered statistically significant.

**Table 4:** Multivariate analysis of social determinants of severe household food insecurity.

Variables		Severe Household Food Insecurity	
		OR (95% CI)	*p* Values *
Household-Head Gender	Male	1	
	Female	2.47 (1.40–4.35)	0.001
Household-Head Education	None/Not complete primary school	1	
	Completed primary school	0.68 (0.52–0.89)	0.006
	Completed secondary school	0.62 (0.44–0.88)	0.007
	Completed college/university	0.65 (0.45–0.92)	0.02
	Religious/informal education only	0.99 (0.70–1.39)	0.95
Household Size		1.03 (1.003–1.06)	0.03
Household Agricultural Activity	No Agricultural Activity	1	
	Livestock Production	0.63 (0.43–0.94)	0.02
	Crop Production	0.59 (0.43–0.80)	0.0009
	Crop and Livestock Production	0.50 (0.36–0.69)	<0.0001
Natural Log of Income Per Household Member in Past 3 Months		0.92 (0.89–0.95)	<0.0001

* *p* value < 0.05 was considered statistically significant. The model is adjusted for regions of Afghanistan.

## Data Availability

All the data, except detailed household size (obtained under strict use permission by the authors from the DIEM), are publicly available and can be accessed here: [https://data-in-emergencies.fao.org/pages/data, accessed on 11 December 2022].
